# Insecticidal and Enzyme Inhibitory Activities of Isothiocyanates against Red Imported Fire Ants, *Solenopsis invicta*

**DOI:** 10.3390/biom10050716

**Published:** 2020-05-05

**Authors:** Yuzhe Du, Michael J. Grodowitz, Jian Chen

**Affiliations:** National Biological Control Laboratory, Biological Control of Pests Research Unit, Agriculture Research Service, United States Department of Agriculture, 59 Lee Road, Stoneville, MS 38776, USA; Yuzhe.du@ars.usda.gov (Y.D.); micheal.grodowitz@ars.usda.gov (M.J.G.)

**Keywords:** isothiocyanates, red imported fire ants, *Solenopsis invicta*, contact and fumigation toxicity, esterase, glutathione *S*-transferase

## Abstract

Contact and fumigation toxicity of four isothiocyanates (ITCs), including allyl isothiocyanate (AITC), 3-butenyl isothiocyanate (3BITC), 3-(methylthio) propyl isothiocyanate (3MPITC) and 2-phenylethyl isothiocyanate (2PEITC), were evaluated against the red imported fire ant worker, *Solenopsis invicta* Buren. 2PEITC and 3MPITC exhibited strong contact toxicity. The median lethal dose (LD_50_)value of AITC, 2PEITC and 3MPITC were 7.99, 2.36 and 2.09 µg/ant respectively. In addition, AITC and 3MPITC also showed strong fumigation toxicity but not 2PEITC. The median lethal concentration (LC_50_) values of AITC and 3MPITC were 32.49 and 57.6 µg/L, respectively. In contrast, 3BITC did not exhibit any contact and fumigation toxicity even at 100 μg/μL. Esterase (EST), glutathione *S*-transferase (GST) and acetylcholinesterase (AChE)-inhibiting activities were assessed for three ITCs in *S. invicta* workers. All three ITCs inhibited both EST and GST activities but not AChE. The in vitro half maximal inhibitory concentration (IC_50_)values of AITC, 2PEITC and 3MPITC for GST were 3.32, 0.61 and 0.66 µg/µL, respectively. These results suggested that naturally occurring ITCs might be potentially useful for developing fire ants control products.

## 1. Introduction

The red imported fire ants, *Solenopsis invicta* Buren, is a significant invasive pest, which was inadvertently introduced into the United States from South America in the 1930s. The current distribution range of *S. invicta* in the United States covers more than 330 million acres in 13 southern and western states and Puerto Rico [1] and they are still spreading northward. This invasive ant causes more than $6 billion annual losses in the United States for damage repair, medical care and control [2].

Current practices for controlling pest ants depend heavily on synthetic insecticides. Although effective, synthetic insecticides have caused public concerns regarding their negative impact, such as resistance development in targeted insects, environmental pollution and effect on human health. To address these issues, a great effort has been made to exploit natural alternates [3]. Naturally occurring compounds are a source of new chemistry for developing control products that are more environmentally friendly. Isothiocyanates (ITCs) are among the compounds emitted by plants of the Brassicaceae in response to insect feeding damage. The toxic effect of several naturally occurring ITCs on insects have been studied, particularly on the fumigation toxicity of ITCs with high volatility, such as methyl isothiocyanate (MITC) and allyl isothiocyanate (AITC). MITC is an effective soil fumigant and AITC is effective for controlling stored-product pest insects [4,5]. AITC is also toxic to the chive gnat, *Bradysia odoriphaga* [6]. In contrast to fumigation toxicity of MITC and AITC, only few naturally occurring ITCs have been evaluated for their contact toxicity against insects. To our knowledge, only eggs of black vine weevil, *Otiorhynchus sulcatus* (F.) have been tested in contact toxicity bioassays [7].

Except for a study on the repellency of microencapsulated AITC to *S. invicta* [8], toxicity of ITCs has never been studied on any pest ants. In our search for naturally occurring insecticidal toxins, four isothiocyanates (ITCs) were identified from Bagrada bug, *Bagrada hilaris* (Burmeister) (Hemiptera: Pentatomidae) using headspace—solid phase microextraction (HS-SPME), including allyl isothiocyanate (AITC), 2-phenylethyl isothiocyanate (2PEITC), 3-butenyl isothiocyanate (3BITC) and 3-(methylthio) propyl isothiocyanate (3MPITC) (Figures S1 and S2; See Supplementary Materials for chemical characterization). In this study, contact and fumigation toxicities of these four naturally occurring ITCs were evaluated against red imported fire ants.

Herbivorous insects have developed several different enzyme systems to detoxify various toxic allelochemicals or xenobiotics from their host plants, including cytochrome P450s (CYP), glutathione *S*-transferases (GSTs) and esterases (EST) [9]. It has been reported that naturally occurring ITCs are good substrates for glutathione *S*-transferase Delta 2(GSTD2) in *Drosophila* [10]. Among the 40 GSTs identified in *Drosophila melanogaster*, the Delta and Epsilon groups are the insect-specific groups, which may have evolved to serve in detoxification and have been associated with insecticide resistance [10]. GSTs can catalyze the conjugation of reduced glutathione (GSH) with electrophilic endogenous and xenobiotic compounds, converting them to less toxic water-soluble products [9,11,12]. GSH is present intracellularly in low concentration and may be conjugated to electrophiles by GSTs [13]. The increased water solubility of the GSH-conjugates then facilitates the excretion of these bound toxins in the urine or feces [14,15]. Several lepidopteran species utilize GSH conjugation for detoxification of GLS-derived ITCs, including African cotton leafworm, *Spodoptera littoralis,* a generalist herbivore [16]. Therefore, in addition to contact and fumigation toxicity, inhibition activities of three active ITCs against esterase α-NA or β-NA (EST α-NA or β-NA), acetylcholinesterase (AChE) and GST in *S. invicta* workers were also assessed.

## 2. Results

### 2.1. Contact Toxicity

Among four ITCs, 2PEITC and 3MPITC exhibited higher contact toxicities than AITC (Table 1) and 3BITC did not cause any mortality at 100 μg/μL. The estimated median lethal dose (LD_50_) values of AITC, 2PEITC and 3MPITC were 7.99, 2.36, 2.09 µg/ant, respectively. Based on LD_50_ values, the contact toxicity of 2PEITC and 3MPITC was about 4 times higher than AITC (Table 1). In contrast, LD_50_ values ranged from 2.17 to 2.58 μg/ant and 1.94 to 2.25 μg/ant for 2PEITC and 3MPITC, respectively, which indicated they have similar contact toxicity.

### 2.2. Fumigation Toxicity

In contrast to its low contact toxicity, AITC has the highest fumigation toxicity among four ITCs. The median lethal concentration (LC_50_) values of AITC and 3MPITC were 32.49 and 57.6 µg/L, respectively. The fumigation toxicity of AITC was about two times higher than 3MPITC (Table 2). However, the LC_50_ value for 2PEITC and 3BITC could not be estimated because of the low mortality at 100 μg/μL.

### 2.3. Inhibition of Esterase, Glutathione S-Transferase, Acetylcholinesterase

**Figure 1:**
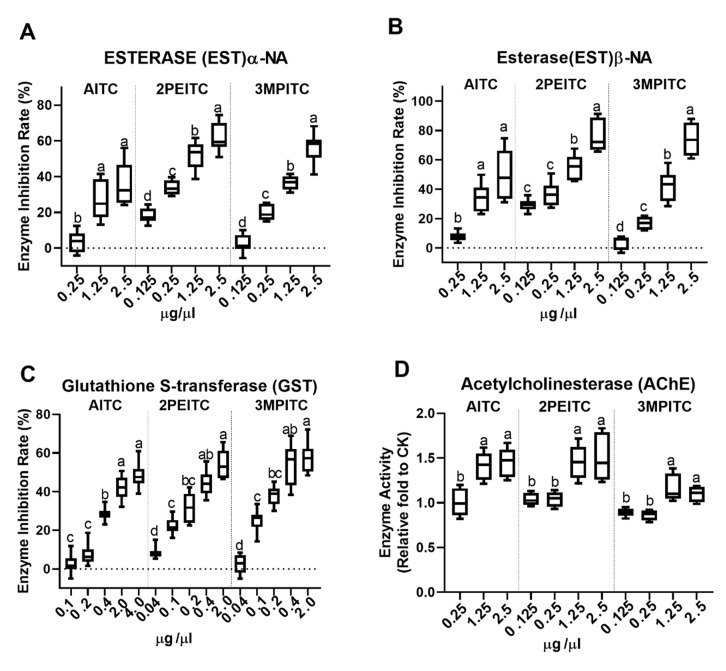
Effect of three isothiocyanates on esterase (EST)α-NA (**A**), esterase (EST)β-NA (**B**), glutathione *S*-transferase (GST) (**C**) and acetylcholinesterase (AchE) (**D**) activities in red imported fire ants, *Solenopsis invicta*. To obtain the homogeneous variance, some percentage of inhabitation rate was arcsine square root transformed. Boxes extends from the 25th to 75th percentiles, with the band indicating median. Means sharing no letter on the top of bars are significantly different, as determined by one-way ANOVA with Tukey’s honestly significant difference (HSD) test and significant values were set at *p* < 0.05.

Because 3BITC could not induce any contact and fumigation toxicity at 100 μg/μL, we only test the enzyme inhibiting activities of other three ITCs. Esterase α-NA or β-NA activities in *S. invicta* workers were reduced by the three ITCs. The inhibition of EST activity was enhanced with the increased doses (Figure 1A,B). The half maximal inhibitory concentration (IC_50_) values of 2PEITC and 3MPITC were 0.95 and 1.87 μg/μL for EST α-NA (Figure 1A) and 0.58 and 1.26 μg/μL for EST β-NA (Figure 1B), respectively. The inhibition rate of AITC reached up to 36.55% and 47.65% for EST α-NA and EST β-NA at 2.5 μg/μL, respectively (Figure 1A,B). Therefore, 2PEITC and 3MPITC were stronger EST activity inhibitors than AITC (Table 3).

GST activity was also inhibited by the three ITCs and IC_50_ values of AITC, 2PEITC and 3MPITC were 3.32, 0.61 and 0.66 μg/μL, respectively (Table 3), which indicated that 2PEITC and 3MPITC were about 5–6 times stronger GST inhibitors than AITC. The inhibition of GST activity was enhanced with the increased dosages of 0.04, 0.1, 0.2, 0.4 μg/μL for 2PEITC and 3MPITC and 0.1, 0.2, 0.4, 2 μg/μL for AITC. However, the inhibition rate reached a plateau at a dosage of 0.4 and 2.0 μg/μL for 2PEITC and 3MPITC and 2.0 and 4.0 μg/μL for AITC (Figure 1C). Both 2PEITC and 3MPITC inhibited EST and GST activity more strongly than AITC (Table 3), which were consistent with their contact toxicities. But they were equally effective in inhibiting GST activity in *S. invicta* workers, while 3MPITC inhibited EST activity less than 2PEITC (Table 3). In addition, three ITCs inhibited GST more strongly than EST at low concentrations (Figure 1A–C).

Inhibition of AChE is one of modes of action of many insecticides, such as organophosphates and carbamates. Therefore, effect of these ITCs on AChE was also investigated. All three ITCs significantly enhanced AChE activities at the concentrations of 1.25 and 2.5 µg/µL but not at 0.125 and 0.25 µg/µL (Figure 1D).

## 3. Material and Methods

### 3.1. Insects

*S. invicta* were collected from Washington County, Mississippi. Ant colonies were maintained in Fluon-coated trays and kept in an insect rearing room at 26 °C. The social form of *S. invicta* colonies was determined using polymerase chain reaction (PCR) on Gp-9 alleles [17]. The PCR results showed all ants used in this study were from monogyne colonies (Figure S3). The colonies were fed with 10% sucrose and frozen house cricket, *Achela domesticus A* and kept at room temperature with ~70% humidity and 16:8 dark: light photoperiod.

### 3.2. Chemicals

**Figure 2:**
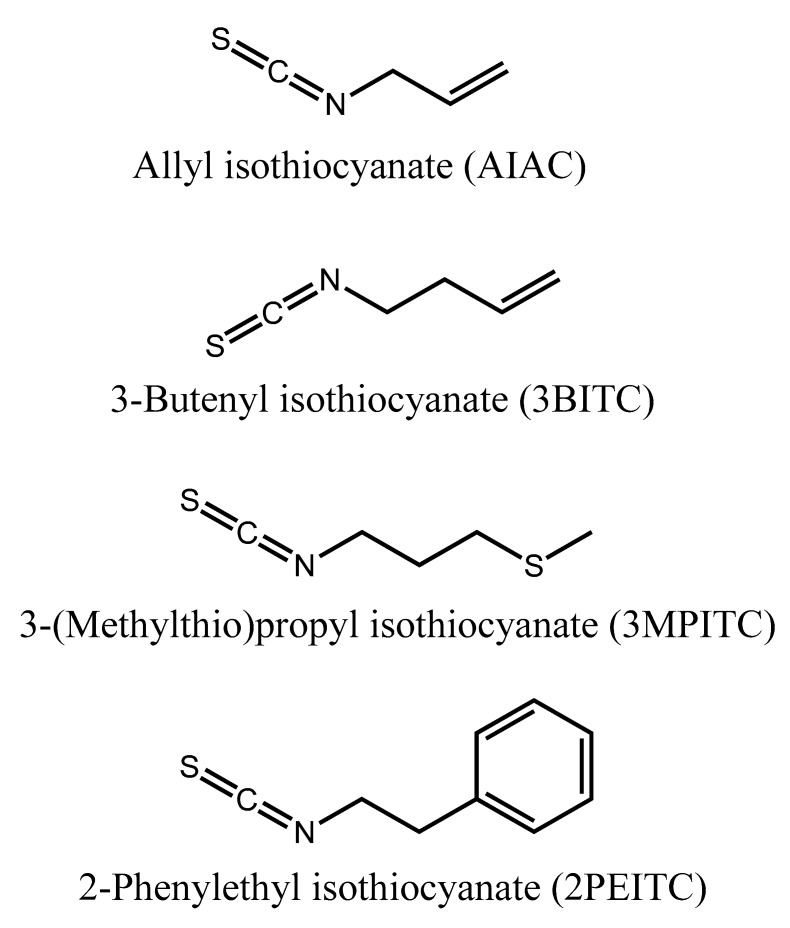
Chemical structures of isothiocyanates found in Bagrada bugs, *Bagrada hilaris.*

Allyl isothiocyanate (AITC), 2-phenylethyl isothiocyanate (2PEITC), 3-butenyl isothiocyanate (3BITC) and 3-(methylthio) propyl isothiocyanate (3MPITC) (Figure 2) were purchased from Sigma-Aldrich (St. Louis, MO, USA). The purities of AITC, 2PEITC,3BITC and 3MPITC were 99.7, 99, 97 and 98%, respectively. The following chemicals were purchased from Sigma-Aldrich as well for enzyme activity assay: Coomassie Brilliant Blue G-250, α-naphthol, β –naphthol, α-naphthyl acetate (α-NA), β-naphthyl acetate (β-NA), β-nicotinamide adenine dinucleotide phosphate (β-NADPH), fast blue B salt, 1-chloro-2,4-dinitrobenzene (CDNB), l-glutathione reduced (GSH), acetylthiocholine iodide (ATC), 5,5’-dithio-bis(2-nitrobenzoic acid) (DTNB).

### 3.3. Contact Toxicity Bioassay

The procedures for contact toxicity bioassay were identical to that described previously [18]. Acetone was used as a solvent. The solution was applied to a worker ant using a 0.749 µl capillary tube. Only large fire ant workers were used in contact bioassay. For AITC, five doses of 4.68, 7.49, 9.36, 14.04, 18.72 μg/ant were used. In contrast, for 2PEITC and 3MPITC, five doses of 0.94, 1.87, 2.81, 3.75, 7.49 μg/ant were used, whereas for 3BITC, only 74.9 μg/ant was used. Acetone alone as a negative control. Treated ants were placed in a capped glass vial and dead ants were counted at 24 h after treatment. There were 10–26 replicates for each dose from at least 3 colonies. A single replicate consisted of 10 ants. Pooled data on 24h mortality was used for calculating LD_50_ values. All bioassays were conducted at the room temperature (~22 °C).

### 3.4. Fumigation Toxicity Bioassay

Fumigation toxicity was evaluated following the previous report with slightly changes [19]. Acetone was used as solvent, a 1 L glass bottle was used as a fumigation chamber and 2 µL test compound was applied in a small plastic tube that was connected to the cap. There were 3 holes (about 1 cm in diameter) on the tube to allow the compound to vaporize into the bottle. Five concentrations were used, including 10, 15, 20, 25, 50 μg/μL for AITC and 10, 25, 37.5, 50, 100 μg/μL for 3MPITC. Only one concentration (100 μg/μL) was used for 2PEITC and 3BITC. Acetone alone was used as a negative control. At least three replicates were used for each combination of compound and colony. Fifty workers were used for each replicate. Mortality at 24 h was used for calculating LC_50_ values. The bioassays were conducted at the room temperature (∼22 °C).

### 3.5. Enzyme Preparation

Whole bodies of *S. invicta* (10 large fire ant workers for EST and GST or 50 heads of large fire ant workers for AChE) were homogenized in ice-cold phosphate buffer (0.1 M, pH 7.0) in a Glass-Col homogenizer. The homogenate was centrifuged (4 °C, 12,000× *g*) in an Eppendorf microcentrifuge for 15 min and the supernatant fraction was then removed by filtration through glass fiber and collected for the following enzyme activity assays immediately. Total protein concentration of each enzyme extraction sample was measured by using a Bradford protein assay kit as described by using bovine serum albumin as a standard [20] (Thermo Scientific., Waltham, MA, USA). Protein content of the enzyme solution was quantified according to the method of Bradford (1976) [20], by using Coomassie Brilliant Blue G-250. Absorbance values were recorded 595 nm using a Thermo Scientific Multiskan Go plate reader.

### 3.6. Inhibition of Esterase, Glutathione S-Transferase, Acetylcholinesterase

For enzyme inhibition testing, three ITCs were prepared by dilution in ethanol, respectively. Five microliters of ITC and 50 μL of 0.04 M (EST) or 0.1M (GST and AchE) sodium phosphate buffer pH 7.0 were mixed in a 96-well microplate. Ethanol without any chemical was treated as a positive control. The original concentrations of ITCs were 0.5, 1, 5% and 10% for EST and AchE and 0.1, 0.25, 0.5, 1 and 5% for GST. Accordingly, the final reaction concentrations of ITCs in each of 96 microplate well were 0.125, 0.25, 1.25 and 2.5 μg/μL for EST and AchE and 0.04, 0.1, 0.2, 0.4 and 2 μg/μL for GST. Five microliters of ITC at different concentrations with 50 μL sodium phosphate buffer and enzyme extract (20 μL for EST and 50 μL for GST and AchE) were incubated for 10 min before the substrate was added. The inhibitory activity (%) of EST or GST was estimated as: (100 – (*V*_max_ of treatment/*V*_max_ of control) × 100).

EST activity against α-NA or β-NA were adapted with minor modification from the assay method of Zhu et al. [21]. Briefly, 20 μL of enzyme solution (diluted 10-fold in 0.04 M sodium phosphate buffer pH 7.0) 5 μL ITCs with 50 μL 0.1M sodium phosphate buffer pH 7.0 and 130 μL of substrate solution (20 μL 0.1 mM α-NA or 0.15 mM β -NA diluted in 110 μL 0.04 M sodium phosphate buffer pH 7.0) were added to each well. A total of 205 μL reaction solution was incubated at 37 °C for 30 min and the reaction was stopped by adding 50 μL fast Blue-SDS (sodium dodecyl sulfate). Absorbance values were recorded at 600 nm or 560 nm for α-NA or β-NA, respectively, using a Multiskan Go plate reader (Thermo Scientific). The esterase activity was calculated based on the standard linear relationship established using α-naphthol or β–naphthol per minute per milligram of protein.

GST activities were determined using CDNB as substrate according to the protocols of Yang et al. [22] with some modifications. The reaction mixture consisted of 50 μL of the enzyme solution (diluted 10-fold in 0.1 M pH 7.0 sodium phosphate buffer), 5 μL ITCs with 50 μL 0.1M sodium phosphate buffer pH 7.0, 10 μL 1.2 mM CDNB, 10 μL 6 mM GSH, totally 125 μL in each of 96 microplate well. Optical density at 340 nm (OD340) was recorded for 10 min at 30 s intervals in a Multiskan Go microplate reader. GST activity was determined using the extinction coefficient of 5.3 mM^−1^ (path length −0.552 cm) for CDNB.

AChE activity was measured using acetylthiocholine (ATC) according to the method of Ellman et al. [23] with some modifications. Each reaction mixture included 50 μL enzyme extract, 5 μL ITCs with 50 μL 0.1M sodium phosphate buffer pH 7.0, 0.75 mM ATC and 0.1 mM DTNB in 100 μL of 0.1 M phosphate buffer pH 7.0, totally 205 μL solution in each of 96 microplate well. The enzyme activity was measured for 10 min at 30 s intervals at 412 nm at room temperature by using a Multiskan Go microplate reader (Thermo Scientific). AChE activities were expressed as nmol ATC hydrolyzed per min per mg protein using the extinction coefficient of 9.19 × 10^4^ M^−1^ cm^−1^. There are three technical replicates and three biological replicates in all the enzyme assay.

### 3.7. Statistical Analysis

Data analyses were performed by SPSS software (IBM SPSS statistics subscription software, Version 26, SPSS Inc., Chicago, IL, USA, 2019). For contact and fumigation toxicity bioassays, the corrected mortality was calculated using Abbott’s formula [24]. The LD_50_ and LC_50_ values with 95% confidence interval (CIs) were estimated by probit analysis. The LC_50_ values were considered as significantly different when the 95% confidence intervals did not overlap. To obtain the homogeneous variance, percent of enzyme inhabitation rate was arcsine square root transformed. The data were normally distributed and had similar variances, then an analysis of variance (ANOVA) followed by Tukey’s honestly significant difference (HSD) post-test to assess significant differences among different concentrations (*p* < 0.05). The IC_50_ values with 95% confidence intervals (CIs) were determined by probit analysis. All statistical analyses were performed by SPSS (version 22.0; SPSS Inc., Chicago, IL, USA).

## 4. Discussion

AITC has been used as a fumigant for many stored product pest insects [25,26,27]. Worfel et al. (1997) demonstrated that AITC reduced the reproduction in *Lasioderma serricorne* in a warehouse and also reduced the activity and reproduction of *Tribolium confusum* [4]. Significant fumigation effects have also been documented for *Tribolium castaneum, Sitophilus zeamais, Sciara coprophila, T. confusum* and other pest insects [27,28,29]. However, the toxicity of AITC differed among developing stages and species of tested insects. In *Bradysia* *odoriphaga,* adult was significantly more sensitive to AITC than the other three developmental stages [6]. According to our bioassay results, AITC has a much higher fumigation toxicity to *S. invicta* (LC_50_: 32.49 μg/L) than other insect species, such as *B. odoriphaga* adults (LC_50_: 7.43 µL/L) [6], *T. castaneum* adults (LC_50_: 4.66 µL/L) [29], *Sitophilus oryzae* adults (LC_50_: 2.7 µL/mL) and *T. confusum* adults (LC_50_: 5.5 µL/mL) [28].

Except for AITC, the LD_50_ values of 2PEITC and 3MPITC are 2.36 and 2.09 µg/ant respectively, which are greatly lower than many other naturally occurring compounds that have been recently reported on *S. invicta*, such as 2-tridecanone (LD_50_: 18.51 to 24.67 µg/ant), formic acid (LD_50_: 124.54 to 197.71 µg/ant) [18] and hexyl benzoate (mean LD_50_: 35.99 µg/ant) [30]. These two compounds may be potentially useful for developing control products that exploit the contact toxicity, such as fire ant mound treatment formulations. The high contact toxicity of 3MPITC and 2PEITC may be due to the methylthio group in 3MPITC and the phenyl group in 2PEITC, respectively. 3BITC did not exhibit any contact and fumigation toxicity although its structure is so similar to AIAC. It will be interesting to investigate the structure and activity relationship for analogs of these ITCs. 2PEITC showed the least fumigation toxicity, which might be due to its low vapor pressure (0.007000 mmHg at 25.00 °C). The vapor pressure was 3.7 mmHg at 30 °C, 2.722 at 25 °C and 0.045 at 25 °C for AITC, 3BITC and 3MPITC, respectively.

Besides the insecticidal activities, all three ITCs exhibited enzyme inhibitory activities to EST and GST as well. All three ITCs inhibited GST at low concentrations and reached plateau at high concentrations. Our results indicated that these three ITCs may be good substrates for GST in *S. invicta,* like in *Drosophila* [10]. As being mentioned before, the important function of GST is detoxification through conjugating reduced GSH with a large number of electrophilic metabolites derived from a variety of xenobiotics, including carcinogens, toxins and drugs [9,11,12]. ITCs are allelochemicals produced by plants to combat insects and other herbivores. The compounds are toxic electrophiles that can be inactivated by being conjugated with intracellular GSH in reactions catalyzed by GSTs [31]. It has also been reported that ITCs inhibited GST in other insect pests. Wadleigh and Yu tested GST activity in response to various ITCs in larvae of fall armyworm, *Spodoptera frugiperda* (J.E. Smith), cabbage looper, *Trichoplusia ni* (*Hübner*), and, velvetbean caterpillar, *Anticarsia gemmatalis* Hübner) using the midgut soluble fraction as the enzyme source [32]. Their results suggested that GST plays an important role in the detoxification of ITCs. All these ITCs were toxic to all tested lepidopterans. They caused acute toxicity in neonates and final-instar larvae [32]. In addition, the activity of GST was inhibited at a low AITC dosage (0.5 μg/mL) in adult *S. zeamais* but was induced at a high AITC dosage (1.5 μg/mL) [27]. Our study also proved that ITCs inhibited GST activity in *S. invicta* at low concentrations but reached a plateau at high concentrations.

Same as GST, EST is another important detoxifying enzymes of insects [9]. Our results indicated that EST (EST α-NA & EST α-NA) in *S. invicta* was inhibited by all three tested ITCs concentrationdependently. And ITCs inhibited GST more strongly than EST at low concentration. However, no tested ITCs inhibited AchE in *S. invicta*. Interestingly, AITC was found to inhibit the AchE activities in *S. zeamais* adults [27]. Further study is needed to understand such differences between *S. invicta* and *S. zeamais* adults.

Our results also indicated that both 2PEITC and 3MPITC inhibited EST and GST activity more strongly than AITC_._ Both 2PEITC and 3MPITC were equally effective for inhibiting GST activity. GST inhibitory activity of ITCs were consistent with their contact toxicity, indicating that inhibition of GST may be one of the modes of action of ITCs against *S. invicta*.

Insect control products based on naturally occurring compounds tend to break down quickly in the environment. They may pose a lower threat to the environment or to human health than the synthetic pesticides. Such products have long been viewed as attractive alternatives to synthetic chemical insecticides for pest management. In our study, the promising contact and fumigant toxicities of some ITCs were demonstrated, indicating these compounds may be useful in developing safer and organic solutions for controlling fire ants in the future.

## Figures and Tables

**Table 1:** Contact toxicity of three isothiocyanates against *S. invicta* workers.

Chemical	LD_50_ (µg/ant)	95% CI^a^ (µg/ant)	Slope ± SEM	*ꭓ*^2 b^ (*df* ^c^, *p*)
AITC	7.99a ^d^	7.48–8.53	6.44 ± 0.41	2.63 (4, <0.001)
2PEITC	2.36b	2.17–2.58	3.00 ± 0.12	42.89 (4, <0.001)
3MPITC	2.09b	1.94–2.25	4.22 ± 0.32	19.35(4, <0.001)

^a^ Confidence Interval. ^b^ Pearson’s chi-squared goodness-of fit test. ^c^ Degree of freedom. ^d^ the same letters within a column are not significantly different when the 95% CI failed to overlap. LD_50_: median lethal dose. SEM: standard error of the mean.

**Table 2:** Fumigation toxicity of two isothiocyanates against *S. invicta* workers.

Chemical	LC_50_ (μg/L)	95% CI^a^ (µg/L)	Slope ± SEM	*ꭓ*^2b^ (*df* ^c^, *p*)
AITC	32.49a^d^	31.39–33.63	7.44 ± 0.50	8.55 (4, 0.56)
3MPITC	57.60b	53.45–62.07	3.56 ± 0.23	112.24 (3, <0.001)

^a^ Confidence Interval. ^b^ Pearson’s chi-squared goodness-of fit test. ^c^ Degree of freedom. ^d^ the same letters within a column are not significantly different when the 95% CI failed to overlap. LC_50_: median lethal concentration.

**Table 3:** IC_50_ values of EST and GST inhibitory activity of three ITCs in *S. invicta.*

Enzyme	Compound	IC_50_ (μg/μL)	95% CI^a^ (μg/μL)	Slope ± SEM	*ꭓ*^2^^b^ (*df* ^c^, *p*)
ESTα-NA	2PEITC	0.95a^d^	0.61–1.47	0.72 ± 0.13	0.68 (2, 0.71)
	3MPITC	1.87a	1.36–2.58	1.26 ± 0.15	4.76 (2, 0.09)
ESTβ-NA	2PEITC	0.58a	0.41–0.82	0.87 ± 0.13	3.97 (2, 0.14)
	3MPITC	1.26b	0.97–1.64	1.71 ± 0.16	6.75 (2, 0.08)
GST	AITC	3.32a	2.09–5.28	0.89 ± 0.10	6.06 (3, 0.1)
	2PEITC	0.61b	0.40–0.92	0.76 ± 0.10	7.24 (3, 0.07)
	3MPITC	0.66b	0.43–1.01	0.76 ± 0.09	20.16 (3, 0.22)

^a^ Confidence Interval. ^b^ Pearson’s chi-squared goodness-of fit test. ^c^ Degree of freedom. ^d^ the same letters within a column for ESTa-NA, ESTβ-NA or GST respectively are not significantly different when the 95% CI failed to overlap. IC_50_: half maximal inhibitory concentration.

## Supplementary Materials

The following are available online at https://www.mdpi.com/2218-273X/10/5/716/s1, Supplementary Materials for Chemical Characterization of Isothiocyanates in Bagrada Bug, *Bagrada hilaris*. Figure S1: Total ion chromatograms of female and male Bagrada bugs in SPME-GC-MS analysis. Figure S2: Mass spectra of four isothiocyanates found in Bagrada bugs. Figure S3: The social form of fire ant, *S.invicta* used in this study.
