# Supplementary material for: Insecticidal and Enzyme Inhibitory Activities of Isothiocyanates against Red Imported Fire Ants, Solenopsis invicta
Source: Biomolecules. 2020 May 5;10(5):716. doi: 10.3390/biom10050716 (PMC7277602; doi:10.3390/biom10050716)
Supplement: Supplementary file 1 [file biomolecules-10-00716-s001.pdf]

Supplemental information

Insecticidal and Enzyme Inhibitory Activities of Isothiocyanates against Red Imported Fire Ants, *Solenopsis invicta*

Yuzhe Du, Michael J. Grodowitz and Jian Chen*

National Biological Control Laboratory, Biological Control of Pests Research Unit, Agriculture Research Service, United States Department of Agriculture, 59 Lee Road, Stoneville, MS38776

Material and Methods

Insects

Bagrada bug, *Bagrada hilaris* Burmeister was fed by broccoli and maintained at the quarantine facility of the Biological Control of Pests Research Unit (BCPRU), USDA-ARS, Stoneville, Mississippi. The original individuals used to establish this colony were obtained near the University of California (Riverside, CA) from London rocket (*Sisymbrium irio* L.) and shortpod mustard (*Hirschfeldia incana* (L.) Lagr.- Foss.) in the fall of 2010.

Headspace-solid-phase microextraction (HS-SPME) of Bagrada bugs

Freeze-thaw method was used to enhance the detection of insect volatiles using HS-SPME [29].

One insect was placed in 20-ml borosilicate glass Volatile Organic Analysis vials and sealed using an open-top cap with EPA standard septa (Davis Instruments, Vernon Hills, IL). The vial with the sample was then tightly caped and sealed with paraffin film and placed in a -80 ^{0}C freezer (Sanyo Scientific North America, Wood Dale, IL) for 10 min. After removing from the freezer, the sample was allowed to thaw for 5 min by dipping the vial in a water bath maintained at 30 ^{0}C. SPME extraction was then performed for 2h at 30 ^{0}C. White SPME fibers (Polyacrylate, 85 μm) and manual holder (Supelco Inc., Bellefonte, PA) were used to collect the volatiles. Before the first time of use, fibers were thermally cleaned at 250 ^{0}C in the injection port of a GC for 12h. The fiber was cleaned for 2h between each sample.

Gas chromatography-mass spectrometry

Compounds on the SPME fiber were analyzed using Gas Chromatography – Mass

Spectrometry (GC-MS). The GC-MS system consisted of an Agilent 7890A gas chromatograph with a DB-5 capillary column (30 m × 0.25 mm i.d., 0.25 μm film thickness) and an Agilent 5975 mass selective detector (Santa Clara, CA). The GC temperature was programmed at an initial temperature of 60 ˚C, then increased to 236 ˚C at a rate of 3 ˚C /min and held for 20 min, and finally increased to 280 ˚C and held for 8 min. Splitless mode was used. The injection temperature was 250 ˚C, and transfer line temperature was 270 ˚C. The mass spectrometer was operated at 70 eV in the electron impact mode. Chemical identification was confirmed by comparing retention times and mass spectra of samples with those of standards. If ITCs standards were not available, identification was made by comparison of their mass spectra with those in either NIST library (National Institute of Standards and Technology, USA) or in the literature.

Result

Isothiocyanate compounds from Bagrada bugs

Four isothiocyanate compounds were identified (Figure 1), including allyl isothiocyanate (AITC), 3-butenyl isothiocyanate (3BITC), 2-phenylethyl isothiocyanate (2PEITC) and 3-(methylthio) propyl isothiocyanate (3MPITC). Their identifications were confirmed using synthetic standards. Chromatograms of male and female Bagrada bugs and mass spectra of all four isothiocyanate compounds are shown in supplemental Figure 1 and 2 respectively.

Discussion

This is the first time that ITC compounds were identified in *B. hilaris*. As we have known, ITC compounds are produced by cruciferous plants (e.g. cabbage, cauliflower and broccoli) to protect them against herbivores. *B. hilaris* is considered a serious economic pest of a variety of cruciferous vegetable crops, a survey of growers from Yuma, AZ, and the Imperial Valley, CA, estimated that > 90% of broccoli acreage planted in 2010 and 2011 was infested with *B. hilaris* at some point in the growing season, and on average, this resulted in stand losses and plant injury exceeding 5 and 10% in cauliflower and broccoli crops, respectively (http://ag.arizona.edu/crops/vegetables/advisories/ more/insect59.html). ITCs in *B. hilaris* are very likely derived from their diets.

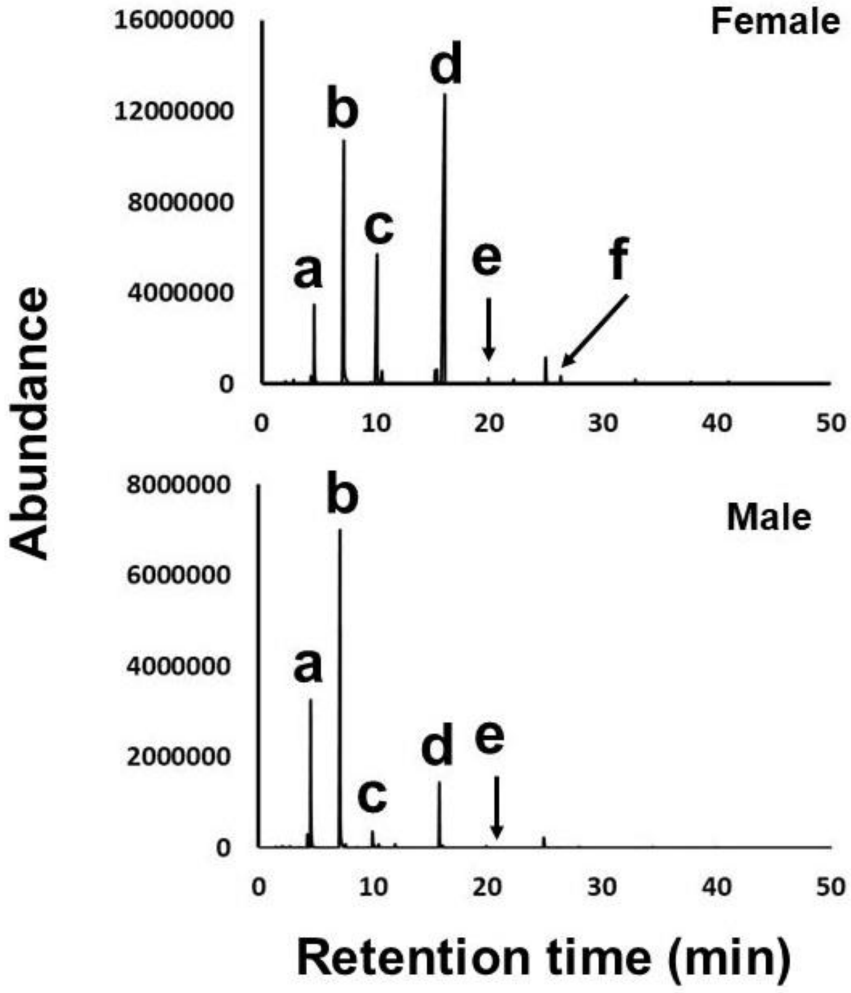

Figure 1. Total ion chromatograms of female and male Bagrada bugs in SPME-GC-MS analysis. Peak assignment: **a**: allyl isothiocyanate (AITC), **b**: 3-buten-1-yl isothiocyanate (3BITC), **c**: trans-2-octen-1-ol, **d**: 1-octen-3-yl acetate, **e**: 3-(methylthio)propyl isothiocyanate (3MPITC), and **f**: 2-phenylethyl isothiocyanate.

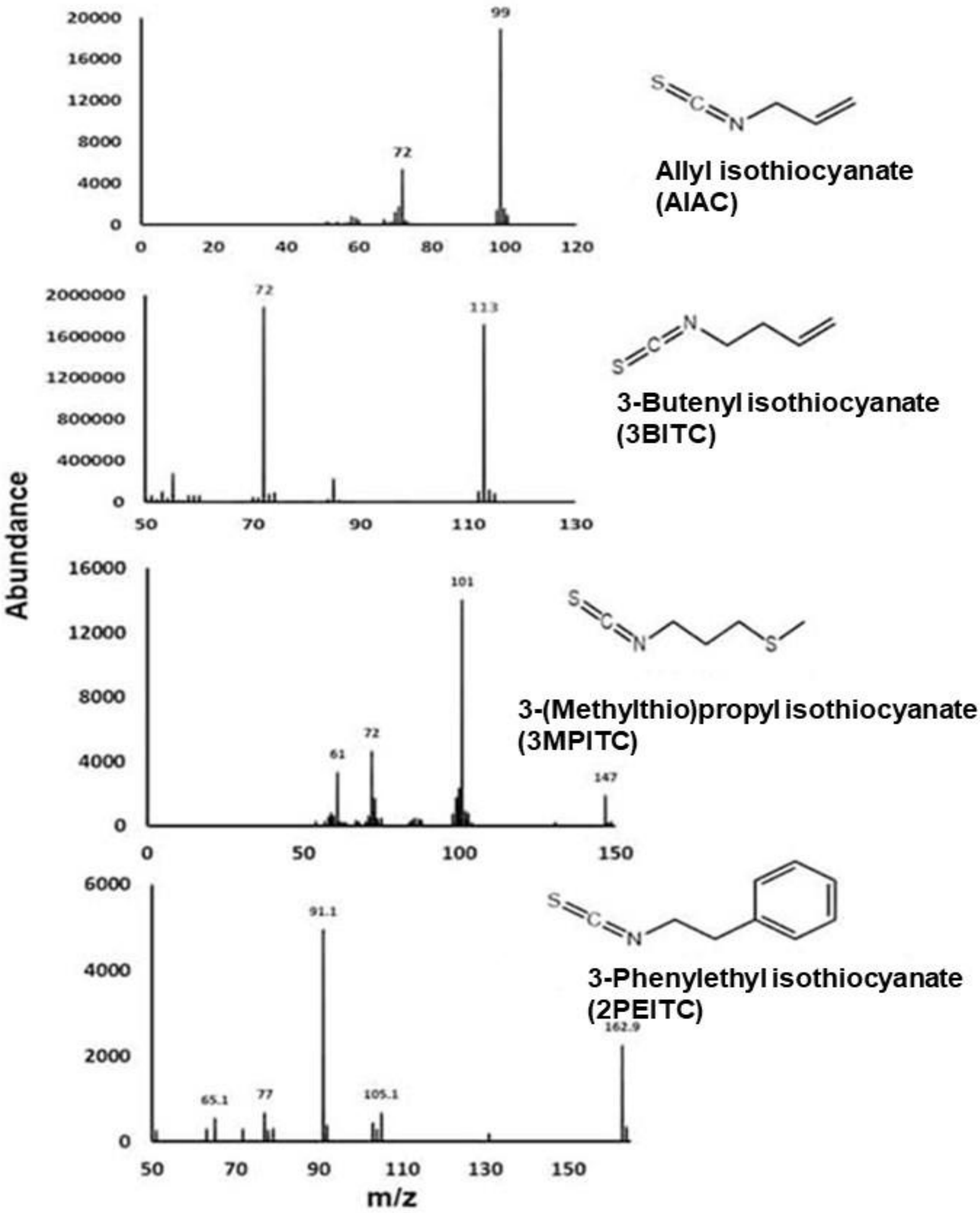

Figure S2. Mass spectra of four isothiocyanates found in Bagrada bugs.

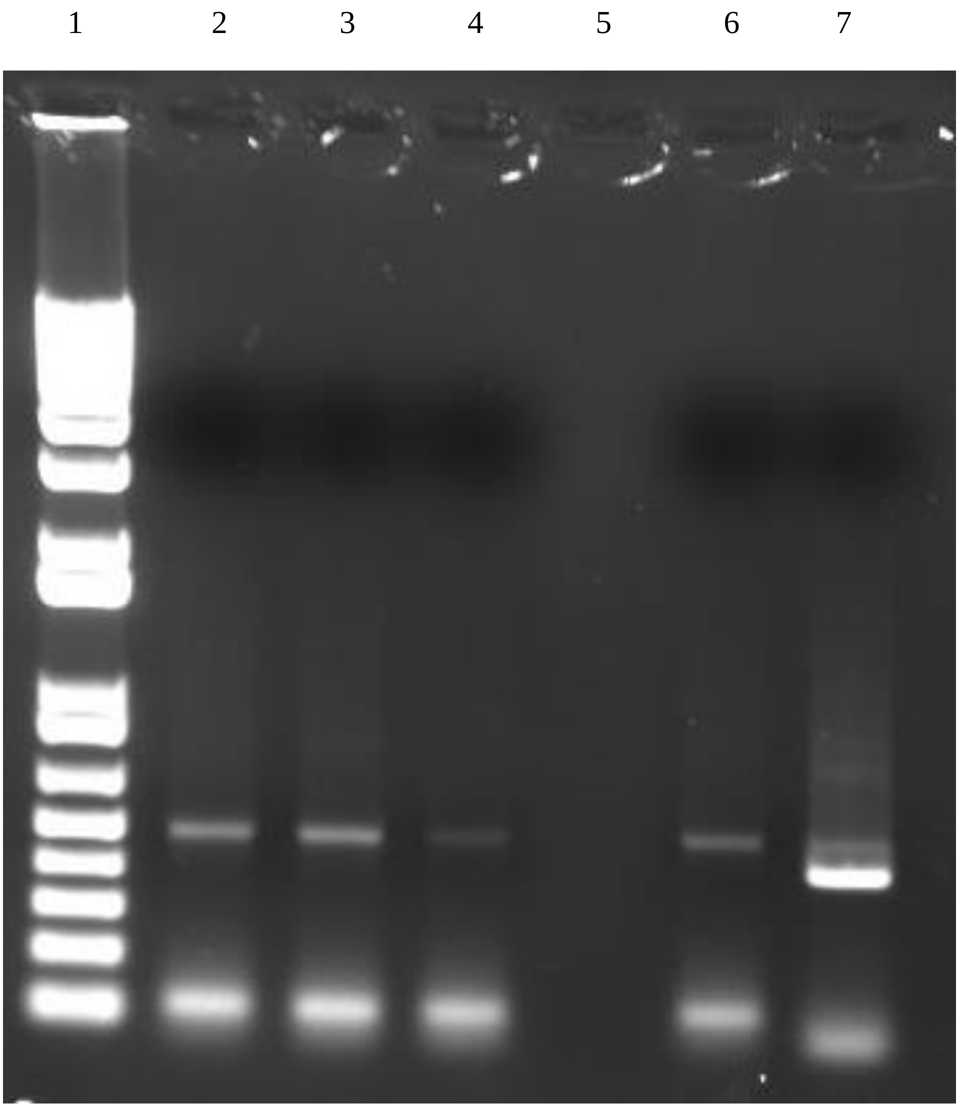

Figure 3. The social form of fire ant, *S.invicta* used in this study. Binding patterns on a 1% agarose gel after multiplex PCR with GP-9 allele-specific primers. Column1, molecular weight markers, Column 2, 3, 4 and 6, PCR conducted with tested *S.invicta* monogyne genomic DNA in this study; Column 7, PCR conducted with a control polygynous genomic DNA.
